# Supplementary material for: Protective Effects of ζ-Carotene-like Compounds against Acute UVB-Induced Skin Damage
Source: Int J Mol Sci. 2023 Sep 12;24(18):13970. doi: 10.3390/ijms241813970 (PMC10530282; doi:10.3390/ijms241813970)
Supplement: Supplementary file 1 [file ijms-24-13970-s001.zip › Supplementary figure legend.pdf]

Supplementary Figure S1. ROS accumulation in HaCAT cells. (A, Band C are NC, UVB and UVB+ CLC groups, respectively).

Supplementary Figure S2. The suppressive effect of CLC on apoptotic factors (A-C) and CPD content (D). (A, B and C are NC, UVB and UVB+ CLC groups, respectively n=3, * p<0.05, ** p<0.01 vs the UVB group.).

Supplementary Figure S3. The body weight (A) and food intake (B) in mice during UVB irradiation. (n=10, 3.2, 3.6, 3.10, 3.15 and 3.20 are March 2, March 6, March 10, March 15 and March 20, respectively).

Supplementary Figure S4. The skin status and clinical score (n=10). (A is NCS group, B is MCS group, C is H-ζS, D is L-ζS, E is VES, F is the clinical evaluation score. ## p<0.01 vs the NCS group and ** p<0.01 vs the MCS group.).

Supplementary Figure S5. A-H are the relative mRNA expression of Keap1/Nrf2/ARE signaling pathway. (n=3, #p< 0.05, ##p < 0.01 vs the NCS group and *p<0.05, **p < 0.001 vs the MCS group, ns is no significance).

Supplementary Figure S6. A-G are the relative mRNA expression of MAPK signaling pathway. (n=3, #p< 0.05, ##p < 0.01 vs the NCS group and *p<0.05, **p < 0.001 vs the MCS group, ns is no significance).

Supplementary Figure S7. The levels of procollagen I (A), HYP (B), HA (C), skin water content (D) and ROS (E) in skin tissue. (n=3, #p< 0.05, ##p < 0.01 vs the NCS group and *p<0.05, **p < 0.001 vs the MCS group, ns is no significance).

Supplementary Figure S8. A-G are the relative mRNA expression of PKA/CREB signaling pathway, H is the tyrosinase activity. (n=3, #p< 0.05, ##p < 0.01 vs the NCS group and *p<0.05, **p < 0.001 vs the MCS group, ns is no significance).
