# Supplementary material for: Protective Effects of ζ-Carotene-like Compounds against Acute UVB-Induced Skin Damage
Source: Int J Mol Sci. 2023 Sep 12;24(18):13970. doi: 10.3390/ijms241813970 (PMC10530282; doi:10.3390/ijms241813970)
Supplement: Supplementary file 1 [file ijms-24-13970-s001.zip › Supplementary File S1.pdf]

Supplementary File

Protective Effects of ζ-Carotene-like Compounds Against Acute UVB-Induced Skin Damage

Liping Zhang[1,2], Shaoxin Liang[2], Zhi Zhang[1,2], Kai Wang[2], Junhan Cao[2], Mengke Yao[2], Ling Qin[2], Changfeng Qu[2*] and Jinlai Miao[2,3,4*]

[1] Department of Special Medicine, School of Basic Medicine, Qingdao University, Qingdao 266071, China

[2] Key Laboratory of Marine Eco-Environmental Science and Technology, First Institute of Oceanography,

Ministry of Natural Resources, Qingdao 266061, China

[3] Qingdao Pilot National Laboratory for Marine Science and Technology, Qingdao 266237, China

[4] Marine Natural Products R&D Laboratory, Qingdao Key Laboratory, Qingdao 266061, China

[*] Correspondence: miaojinlai@fio.org.cn, cfqu@fio.org.cn; Tel: +86 532 88967430, +86 532 88966322

S1. **Methods**

S1.1 Antioxidant Enzyme Activities

The same kit was used for cell and animal experiments. The number of cells was 5 million, and the animal sample tissue was 0.1g.

SOD: The manual is available at https://www.solarbio.com/goods-9124.html (access dates is June 2, 2023)

The order and quantity of adding samples are shown in Table S3.

Table S3. SOD sample determination of the sample list.

Reagent Name (μL)	Measuring	Control	Blank 1	Blank 2
Sample	90	90	0	0
Reagent 1	240	240	240	240
Reagent 2	60	0	60	0
Reagent 3	180	180	180	180
H_2O	400	460	490	550
Reagent 4	30	30	30	30

CPX: The manual is available at https://www.solarbio.com/goods-9204.html (access dates is June 2, 2023)

The order and quantity of adding samples are shown in Table S4.

Table S4. GPX sample determination of sample list.

Reagent Name (μL)	Measuring Tube	Control Tube
Sample	20	0
Reagent 1	20	20
	37°C, 5min	
Reagent 2	10	10
	37°C, 5min	
Reagent 3	200	200
Sample	0	20

After thorough mixing, the mixture was centrifuged at 4000 rpm for 5 min at room temperature, and the supernatant was removed into the previous EP tube.

Reagent Name (μL)	Measuring	Control	Standard	Blank
Diluent	0	0	0	100
Supernatant	100	100	0	0
Reagent 4	100	100	100	100
Reagent 5	25	25	25	25

CAT: The manual is available at https://www.solarbio.com/pdf/2021-9-27/BC0205.pdf (access dates is June 2, 2023)

The order and quantity of adding samples are shown in Table S5.

Table S5. CAT sample determination of sample list.

Reagent Name (μL)	Amount of Sample Added
Sample	10
Working Solution	190

MDA: The manual is available at https://www.solarbio.com/images/202308/1691397344398226369.pdf (access dates is June 2, 2023)

The order and quantity of adding samples are shown in Table S6.

Table S6. MDA sample determination of sample list.

Reagent Name (μL)	Measuring	Blank
Working Solution	300	300
H_2O	0	100
Sample	100	0
Reagent 3	100	100

S1.2 HYP and Tyrosinase Activity

HYP: The manual is available at https://www.solarbio.com/pdf/2021-9-27/BC0255.pdf (access dates is June 2, 2023)

The order and quantity of adding samples are shown in Table S7.

Table S7. MDA sample determination of sample list.

Reagent Name (μL)	Blank	Measuring	Standard
Sample		60	
Standard			60
Reagent 1	60	60	60
The mixture was mixed and allowed to stand for 20 min at room temperature.			
Reagent 2	60	60	60
H_2O	180	120	120
The mixture was mixed, bathed in water for 20 min at 60 ° C, removed and allowed			

to stand at room temperature for 15 min, and 200 μL was placed in a microglass cuvette/96-well plate for A_{560} detection.

Tyrosinase Activity: The manual is available at https://www.solarbio.com/images/202306/1686897704035891453.pdf (access dates is June 2, 2023)

The order and quantity of adding samples are shown in Table S8.

Table S8. Tyrosinase activity sample determination of sample list.

Reagent Name (μL)	Measuring
Reagent 1	180
Sample	20

S1.3 SA-β-Gal

The manual is available at https://www.solarbio.com/images/202307/1689917727663033206.pdf (access dates is June 2, 2023)

The steps were as follows:

1. For cells cultured in 6-well plates, the cell culture medium was removed via aspiration, washed once with PBS, added to 1 mL β-gal fixative, and fixed at room temperature for 15 min. For other types of culture plates, the amount of fixative and subsequent solution was determined according to this ratio.

2. The cell fixative was removed through aspiration and the cells were washed 3 times with 1×PBS for 3 min each time.

3. The staining solution was configured in proportion. The β-Gal cleaning solution was removed through aspiration, and 1ml of staining working solution was added to each well.

4. The solution was incubated overnight at 37 °C, and for this the letter or a wrapping live 6-orifice plate membrane could be used to prevent evaporation.

5. The solution was observed under an ordinary light microscope.

S1.4. ROS Fluorescence Detection

ROS: The manual is available at https://www.solarbio.com/pdf/2020-

shsj/D6470.pdf (access dates is June 2, 2023)

The steps were as follows:

1. Make 1-10 mM DMSO stock solution. The unused DMSO stock solution should be aliquoted into a single use vial and stored at -20 °C. Keep from light.

2. Make the dye at a working concentration of 1–10μM in a physiological buffer (such as PBS, HBSS, HEPES). The optimal working concentration for your application must be empirically determined.

3. Remove cells from growth media, add the dye working solution (from Step 2) to the cells, and incubate the cells at RT or 37 °C for 5 to 60 minutes.

4. Remove the dye working solution, wash with pre-warmed HBSS and add pre-warmed HBSS or growth medium and incubate at the optimal temperature. The optimal recovery time can vary widely, as some cell types normally exhibit low levels of esterase activity.

5. Determine the baseline fluorescence intensity of a sample of the loaded cells prior to exposing the cells to experimental inducements.

S1.5 Elisa Detection

The TNF-α, NF-κB, IL-6, IL-β, HA, CPD, ROS and PC-1 levels were measured using an enzyme-linked immunosorbent assay kit (double antibody one-step sandwich).

The steps were as follows:

1. Remove the required slats from the aluminum bag after 20 min equilibrium at room temperature.

2. Set standard wells and sample wells and add different concentrations of standard 50 μL to each standard hole.

3. First add 10 uL of the sample to the sample wells to be tested, then 40 uL of the sample diluent. Blank wells were not added.

4. In addition to blank wells, 100 mL of horseradish peroxidase (HRP)-labeled detection antibody was added to each well of the standard and sample wells, and the reaction wells were sealed with the sealing plate membrane and incubated in a 37 °C water bath or incubator for 60 min.

5. The liquid was discarded, and each well patted dry with absorbent paper, filled

with washing liquid and left for l min. The washing liquid was shaken off, and the wells patted dry with absorbent paper. This was repeated 5 times to wash the board.

6. Then, 50 L quantities of substrate A and B were added to each well and incubated at 37 ° C in the dark for 15 min.

7. Then, 50μL of termination solution was added to each well, and within 15 min the value of each well was measured at a wavelength of OD_{450nm}.
