# Supplementary material for: Protective Effects of ζ-Carotene-like Compounds against Acute UVB-Induced Skin Damage
Source: Int J Mol Sci. 2023 Sep 12;24(18):13970. doi: 10.3390/ijms241813970 (PMC10530282; doi:10.3390/ijms241813970)
Supplement: Supplementary file 1 [file ijms-24-13970-s001.zip › Supplementary tables.pdf]

Supplementary Table S1. Primers gene sequence in animal experiments.

Gene name	Primer fowerd 5’-3’	Primer reversed 5’-3’
Nrf2	TTCCTTCAGCAGCATCCTCTCC	AATCTGTGTTGACTGTGGCATCTG
HO-1	CTGCGTTCCTGCTCAACATCC	GGGCTCTGGTCCTTGGTGTC
NQO1	CTGCGTTCCTGCTCAACATCC	GGGCTCTGGTCCTTGGTGTC
COX-2	GGTTGCTGGTGGTAGGAATGTTC	TCTGCCTGCTCTGGTCAATGG
P-38	GATACAAAGACGGGGCATCG	CACGATGTTGTTCAGGTCCG
ERK	GCCTTCCAACCTCCTGCTGAAC	CGTACTCTGTCAAGAACCCTGTGTG
JNK	CGCCTTATGTGGTGACTCGCTAC	CTCCCATGATGCACCCAACTGAC
MMP-1	ATTCTACTGATATCGGGGCTTTGA	ATGTCCTTGGGGTATCCGTGTAG
MMP-2	ACCATGCGGAAGCCAAGATGTG	AGGGTCCAGGTCAGGTGTGTAAC
collagen type I	AGGGCAAAGATCCCTGAAGT	GGCAGAAAGCACAGCACTC
AP-1	CTTCTACGACGATGCCCTCAACG	GCCAGGTTCAAGGTCATGCTCTG
PKA	GAGCAGGAGAGCGTGAAAGAG	GGGCTGTATTCTGAGAAGGGG
CREB	CCAGTCTCCACAAGTCCAAACA	GGCACTGTTAGAGTGGTGGTATG
MITF	AGAAGCTGGAGCATGCGAACC	GTTCCTGGCTGCAGTTCTCAAG
TYR	AGTCGTATCTGGCCATGGCTTCTTG	GCAAGCTGTGGTAGTCGTCTTTGTC
TYRP1	CTGCGATGTCTGCACTGATGACCTG	TTTCTCCTGATTGGTCCACCCTCAG
TYRP2	GGGCAGCGAGACCAGACGAT	TTGGCAATTTCATGCTGTTTCTTC
Rab27A	GTAGTGAAAGAGGAGGAAGC	TCATTATCAGGTCCAGAAGC
IL-6	TTCGGTCCAGTTGCCTTCTCC	TTCTGAAGAGGTGAGTGGCTGTC
IL-β	TGGCTTATTACAGTGGCAATGAGG	AGTGGTGGTCGGAGATTCGTAG

NF-KB	CTCTCGCCTGCCTCCACAAG	ATGTCTCCACGCCGCTGTC
TNF-α	CTCATCTACTCCCAGGTCCTCTTC	CGATGCGGCTGATGGTGTG
GAPDH	AGGTCGGTGTGAACGGATTTG	GGGGTCGTTGATGGCAACA

Supplementary Table S2. Primers gene sequence in cell experiments.

Gene name	Primer fowerd 5’-3’	Primer reversed 5’-3’
Nrf2	CAGTGCTCCTATGCGTGAA	GCGGCTTGAATGTTTGTC
Keap1	TGCTCAACCGCTTGCTGTATGC	GCGAGAAGAGCCCTGATTGTACTG
HO-1	ACAGATGGCGTCACTTCG	TGAGGACCCACTGGAGGA
NQO1	AGGATGGGAGGTACTCGAATC	TGCTAGAGATGACTCGGAAGG
PKA	GAGCAGGAGAGCGTGAAAGAG	GGGCTGTATTCTGAGAAGGGG
CREB	CCAGTCTCCACAAGTCCAAACA	GGCACTGTTAGAGTGGTGGTATG
MITF	AGAAGCTGGAGCATGCGAACC	GTTCCTGGCTGCAGTTCTCAAG
TYR	AGTCGTATCTGGCCATGGCTTCTTG	GCAAGCTGTGGTAGTCGTCTTTGTC
TYRP1	CTGCGATGTCTGCACTGATGACCTG	TTTCTCCTGATTGGTCCACCCTCAG
TYRP2	GGGCAGCGAGACCAGACGAT	TTGGCAATTTCATGCTGTTTCTTC
Rab27A	GTAGTGAAAGAGGAGGAAGC	TCATTATCAGGTCCAGAAGC
GAPDH	AGGTCGGTGTGAACGGATTTG	GGGGTCGTTGATGGCAACA
