# Supplementary figures and images for: Protective Effects of ζ-Carotene-like Compounds against Acute UVB-Induced Skin Damage
Source: Int J Mol Sci. 2023 Sep 12;24(18):13970. doi: 10.3390/ijms241813970 (PMC10530282; doi:10.3390/ijms241813970)

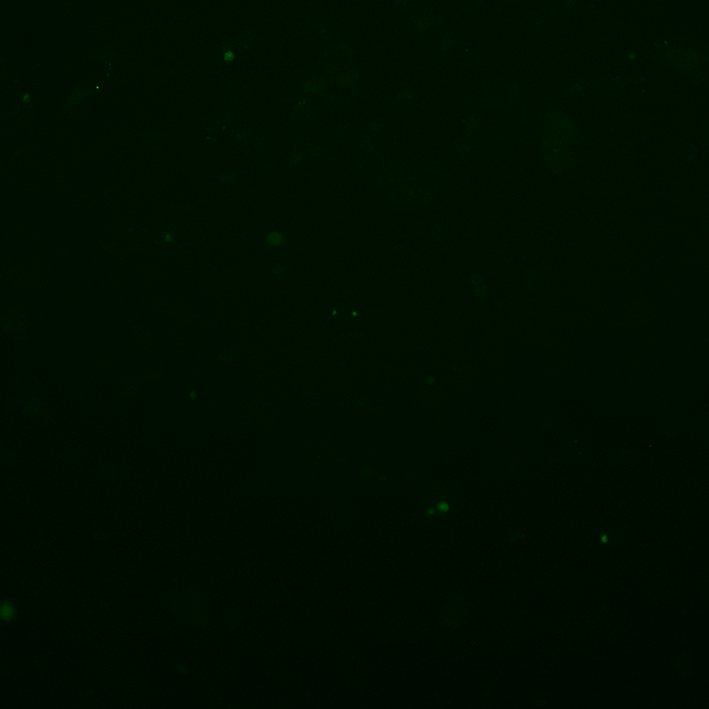

Supplement: Supplementary file 1 [file ijms-24-13970-s001.zip › Figure S1A.tif]

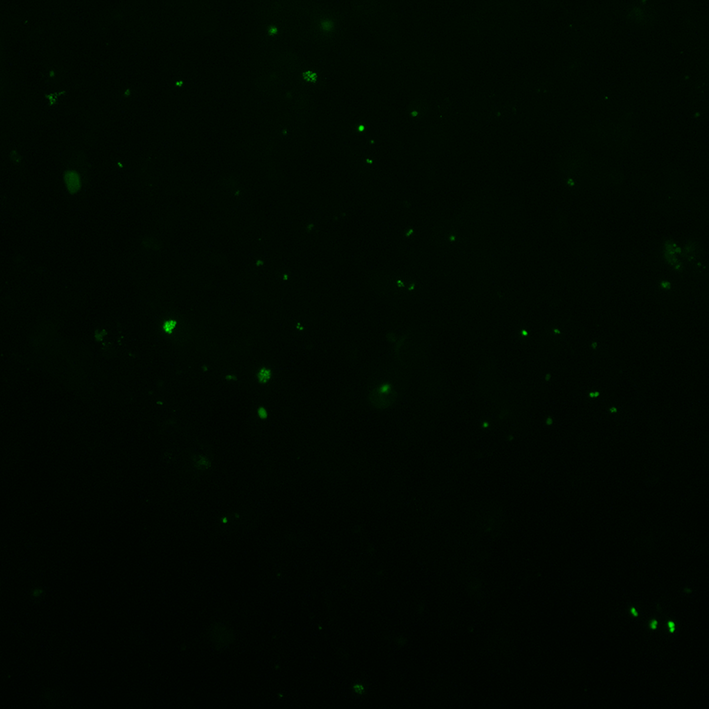

Supplement: Supplementary file 1 [file ijms-24-13970-s001.zip › Figure S1B.tif]

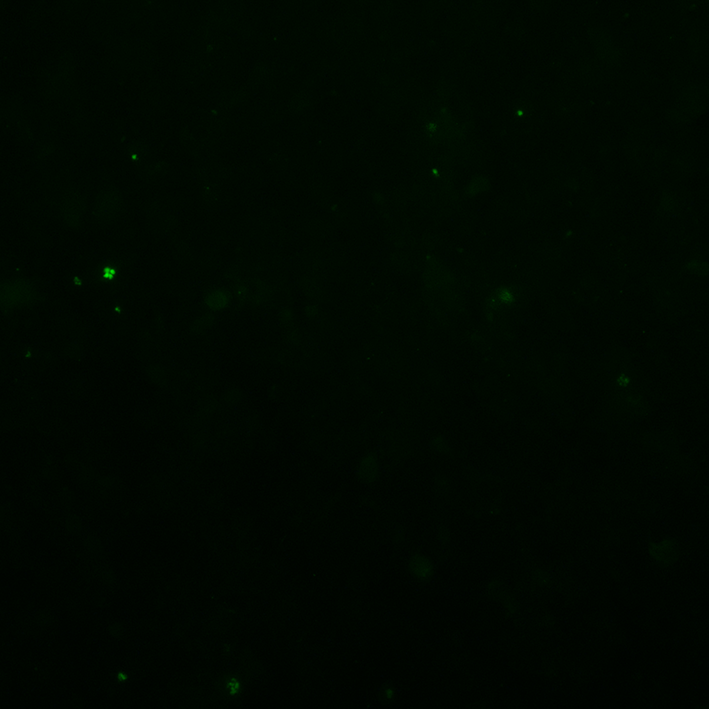

Supplement: Supplementary file 1 [file ijms-24-13970-s001.zip › Figure S1C.tif]

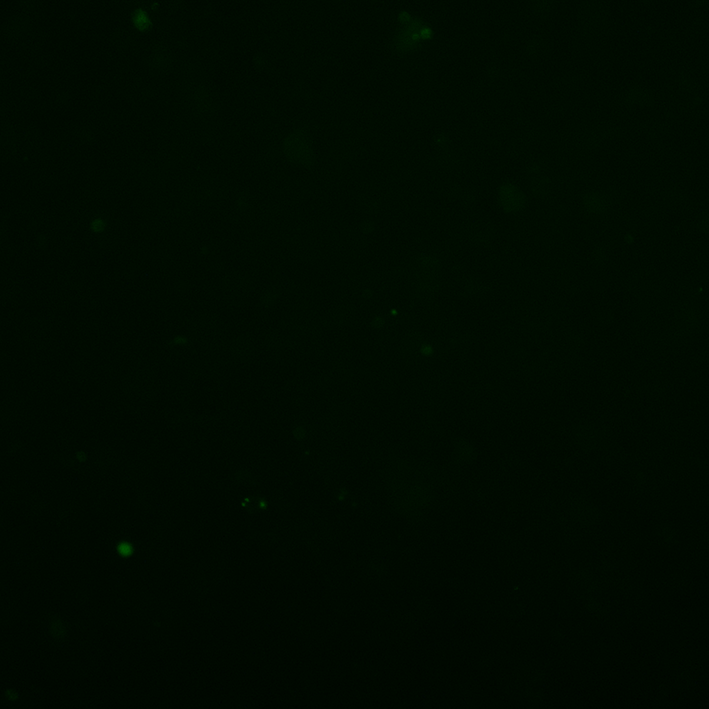

Supplement: Supplementary file 1 [file ijms-24-13970-s001.zip › Figure S2A.tif]

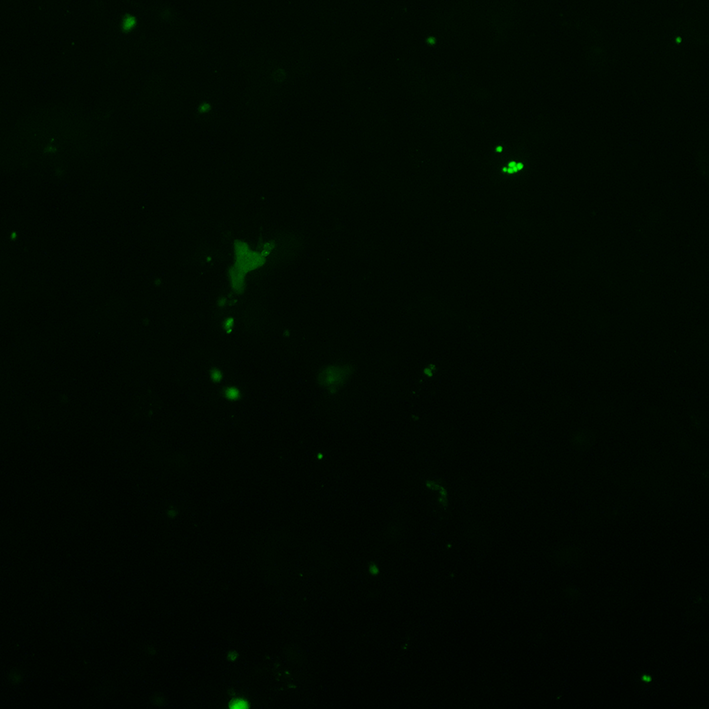

Supplement: Supplementary file 1 [file ijms-24-13970-s001.zip › Figure S2B.tif]

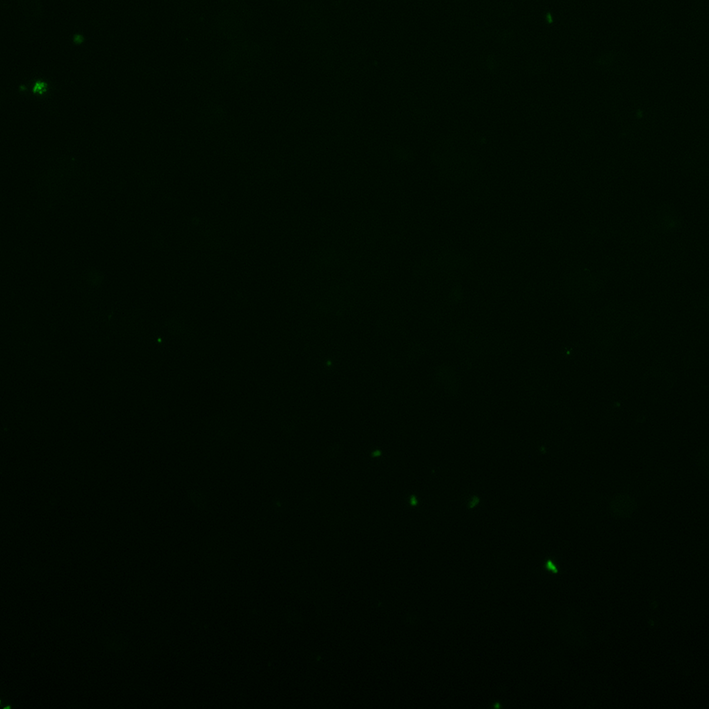

Supplement: Supplementary file 1 [file ijms-24-13970-s001.zip › Figure S2C.tif]

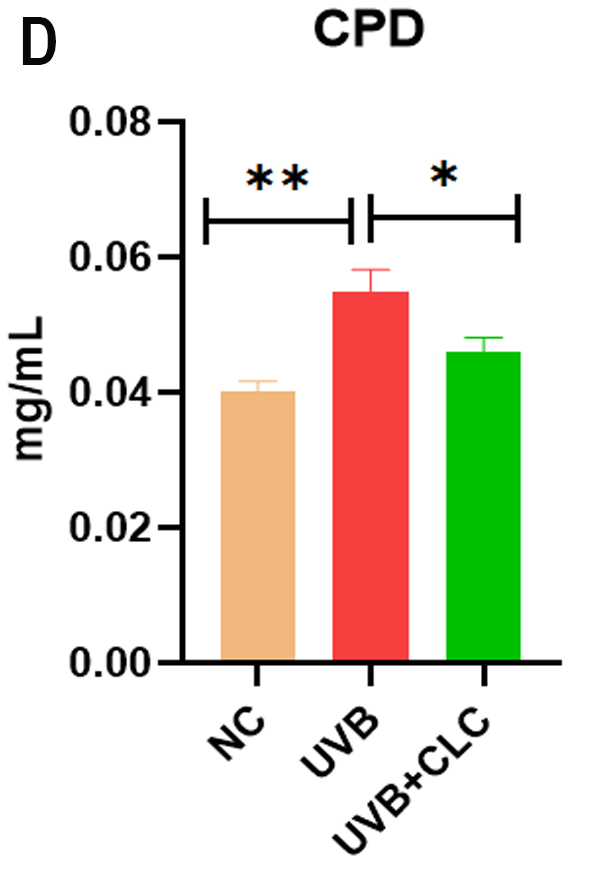

Supplement: Supplementary file 1 [file ijms-24-13970-s001.zip › Figure S2D.tif]

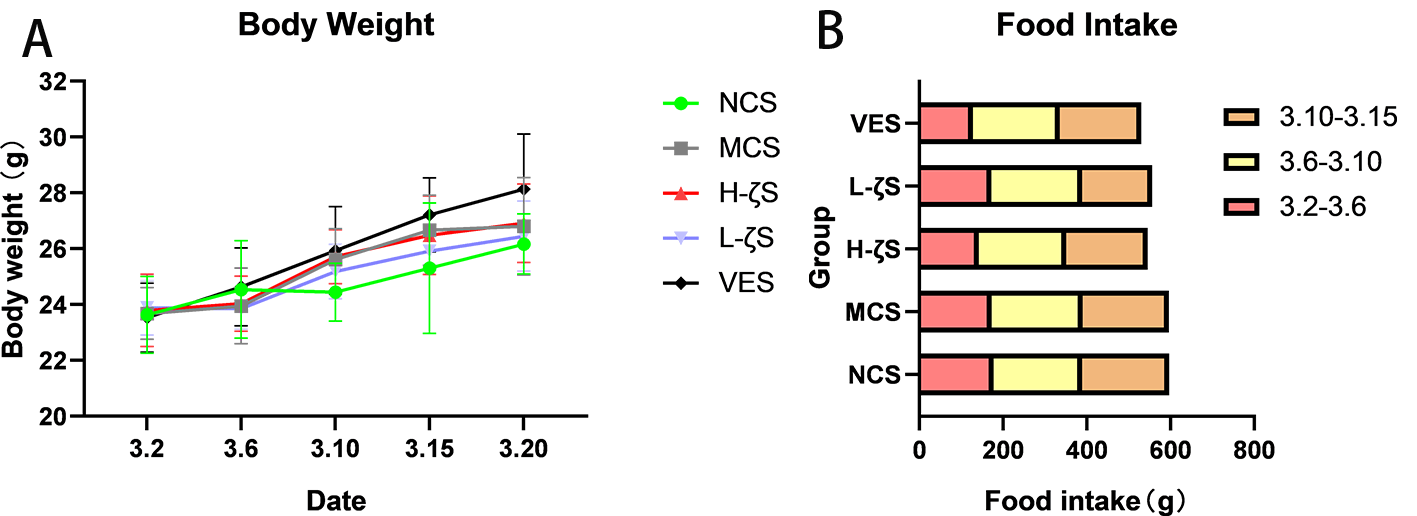

Supplement: Supplementary file 1 [file ijms-24-13970-s001.zip › Figure S3.tif]

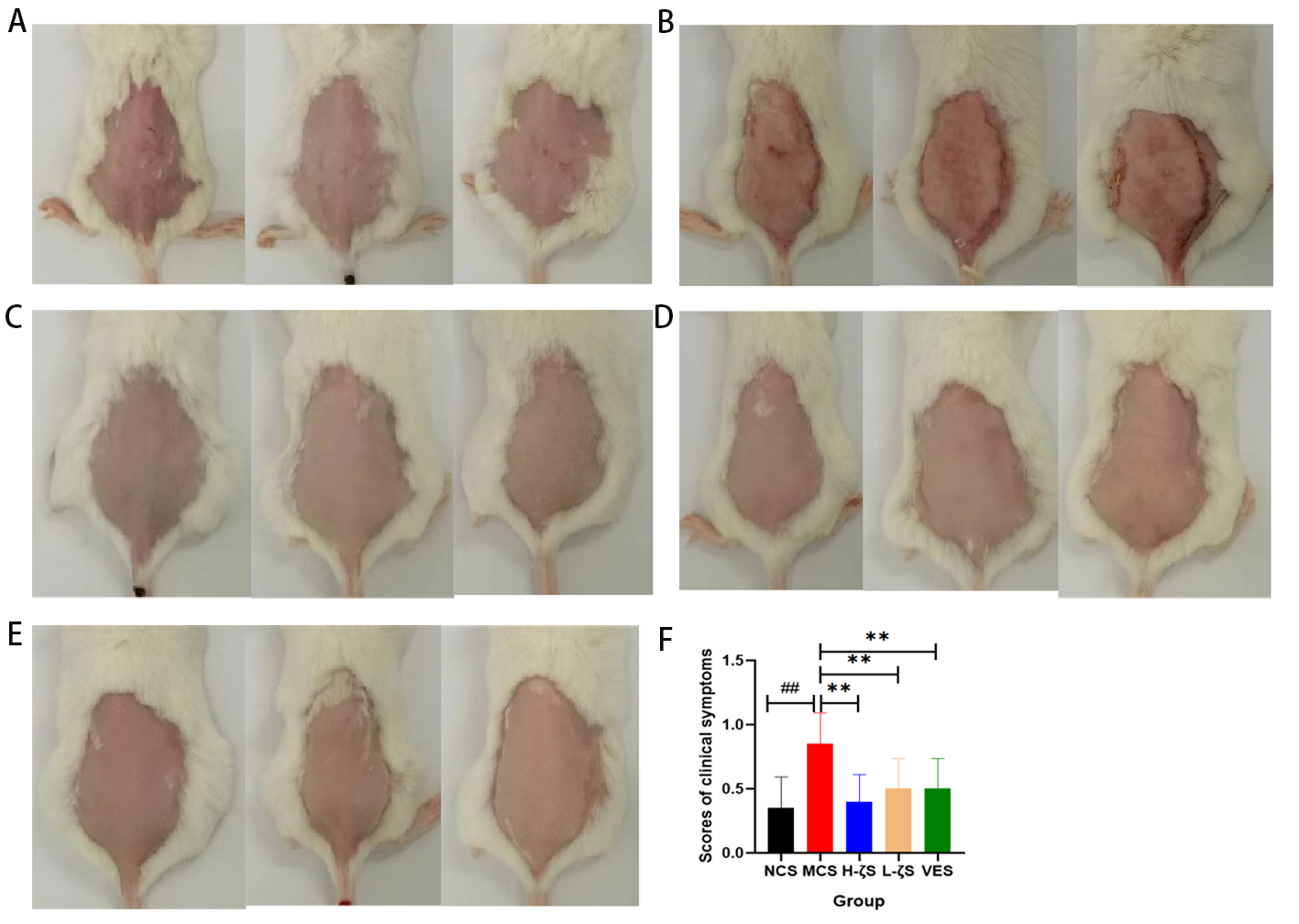

Supplement: Supplementary file 1 [file ijms-24-13970-s001.zip › Figure S4.tif]

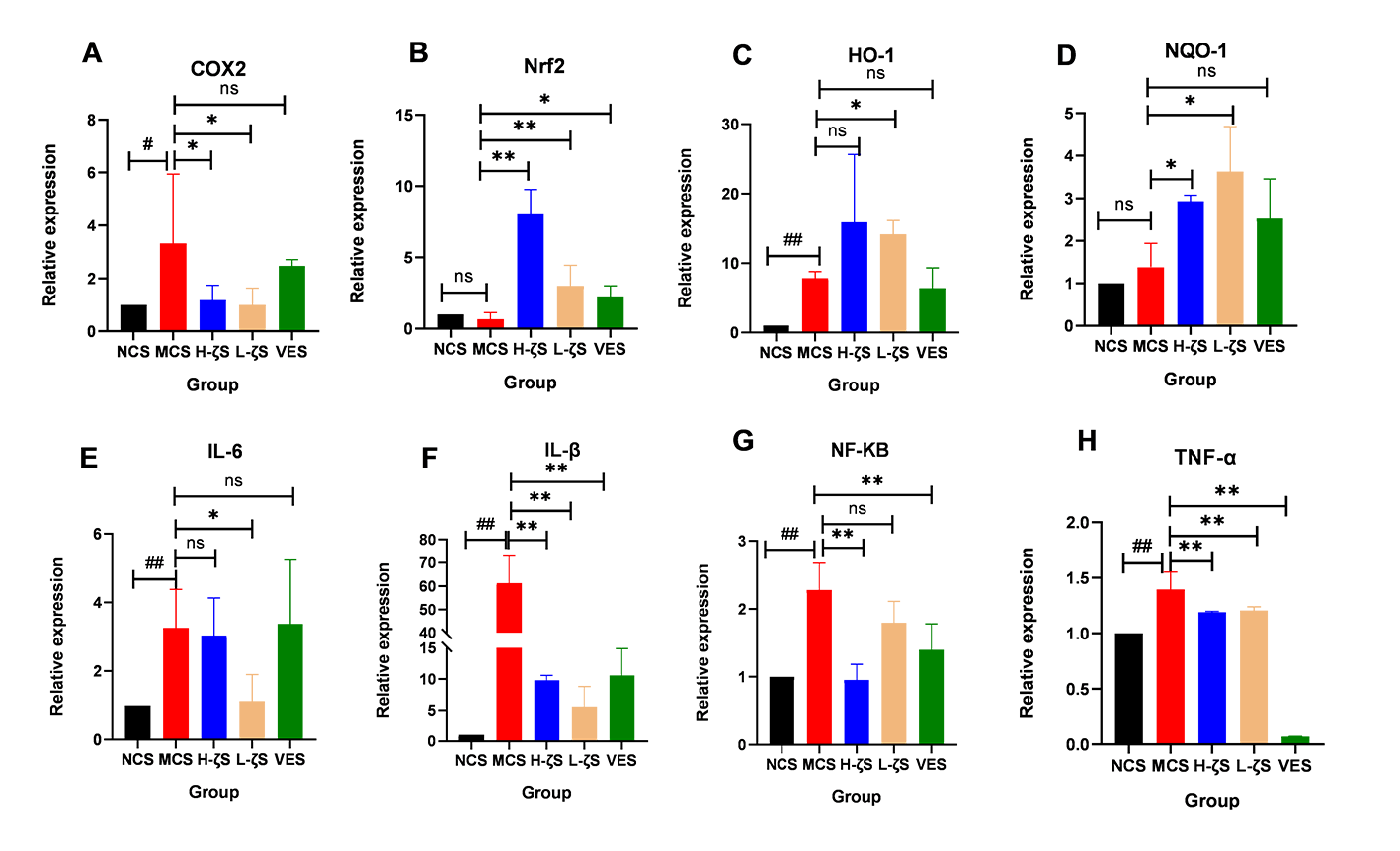

Supplement: Supplementary file 1 [file ijms-24-13970-s001.zip › Figure S5.tif]

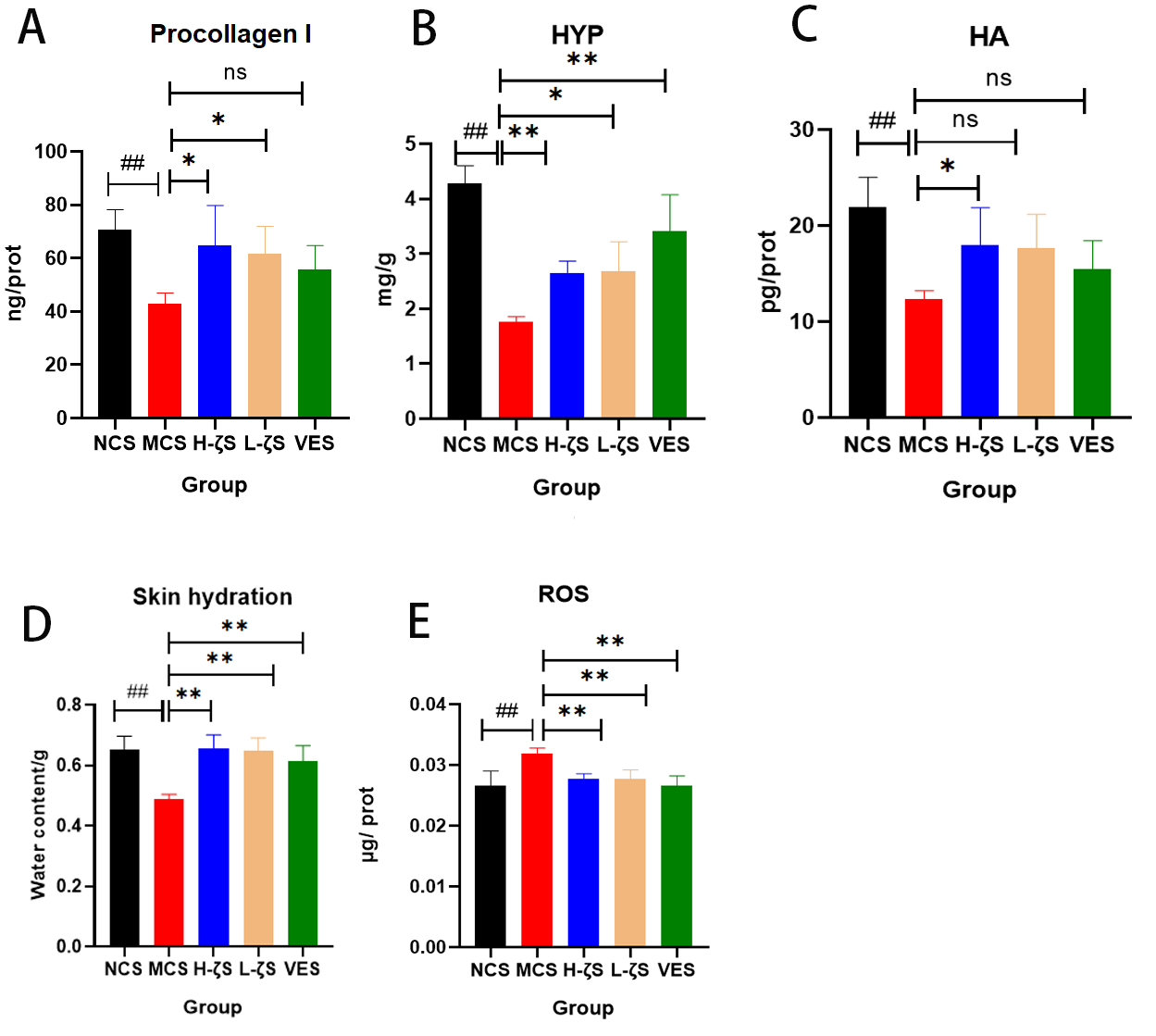

Supplement: Supplementary file 1 [file ijms-24-13970-s001.zip › Figure S7.tif]

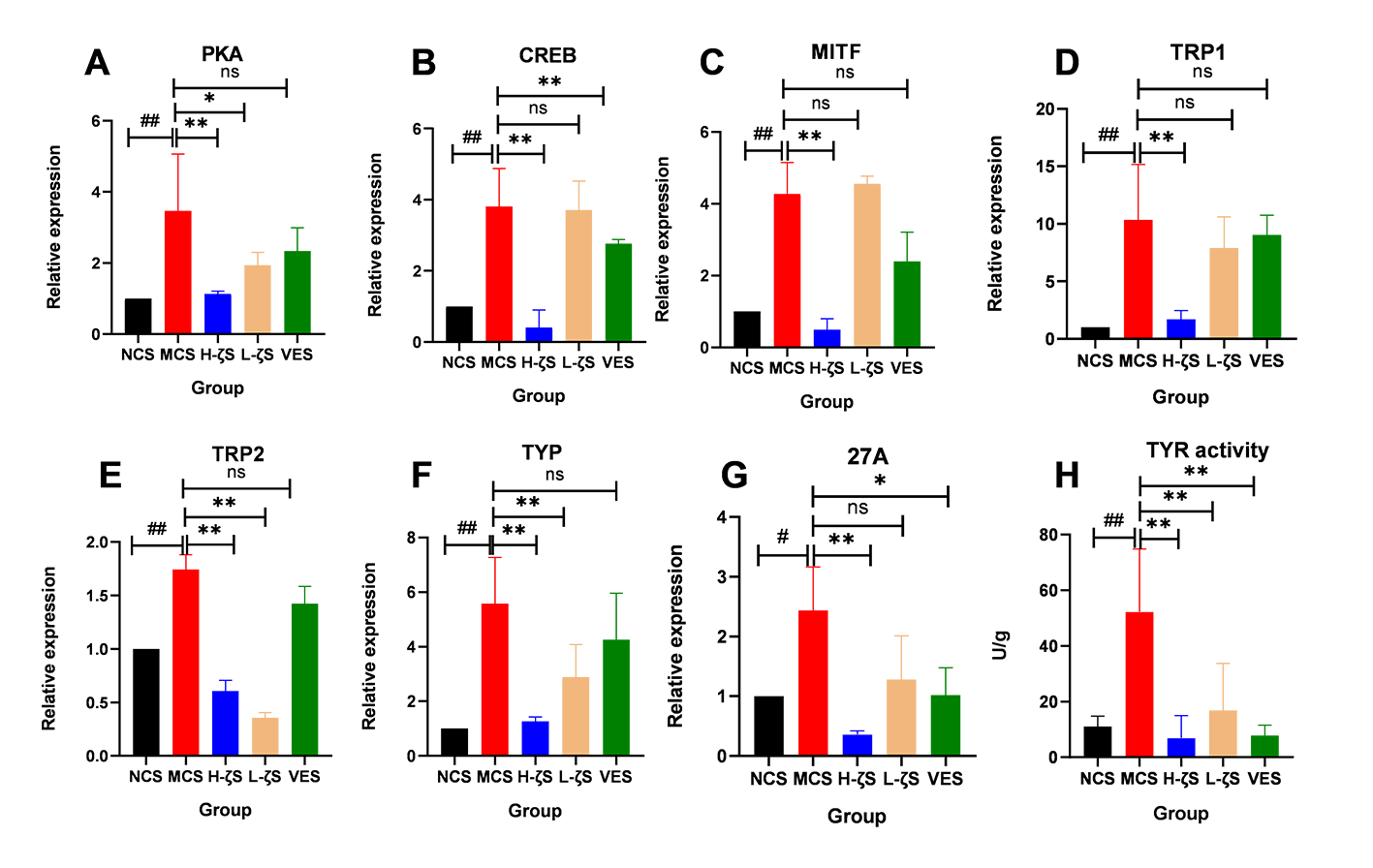

Supplement: Supplementary file 1 [file ijms-24-13970-s001.zip › Figure S8.tif]

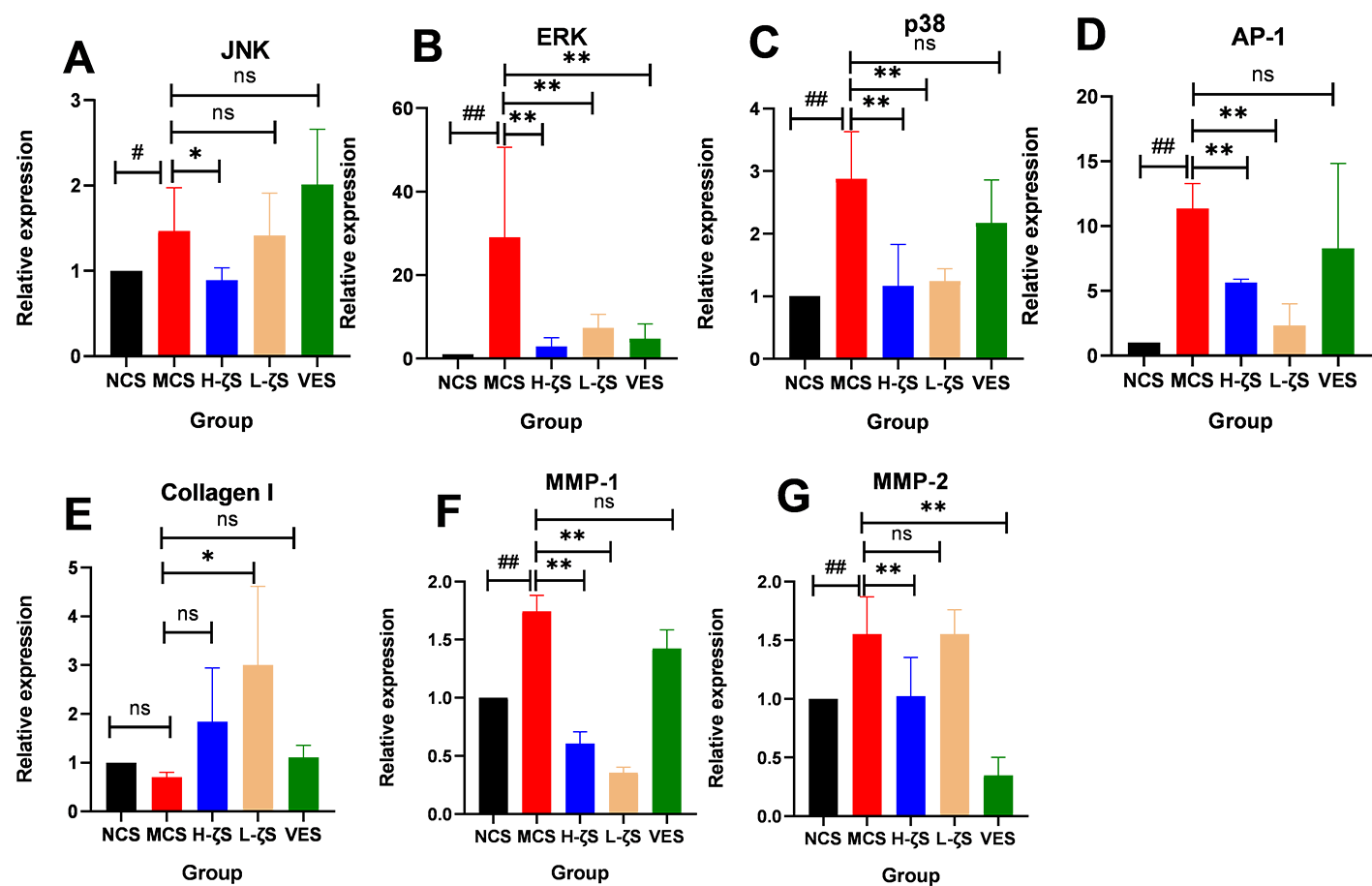

Supplement: Supplementary file 1 [file ijms-24-13970-s001.zip › FigureS6.tif]
